# Key Determinants of Cell-Mediated Immune Responses: A Randomized Trial of High Dose Vs. Standard Dose Split-Virus Influenza Vaccine in Older Adults

**DOI:** 10.3389/fragi.2021.649110

**Published:** 2021-05-21

**Authors:** Chris P. Verschoor, Laura Haynes, Graham Pawelec, Mark Loeb, Melissa K. Andrew, George A. Kuchel, Janet E. McElhaney

**Affiliations:** ^1^ Health Sciences North Research Institute, Sudbury, ON, Canada; ^2^ Northern Ontario School of Medicine, Sudbury, ON, Canada; ^3^ UConn Center on Aging, University of Connecticut School of Medicine, Farmington, CT, United States; ^4^ Department of Immunology, University of Tübingen, Tübingen, Germany; ^5^ Department of Pathology and Molecular Medicine, McMaster University, Hamilton, ON, Canada; ^6^ Department of Medicine (Geriatrics), Dalhousie University, Halifax, NS, Canada

**Keywords:** cell-mediated immune responses, high-dose vs. standard-dose split-virus influenza vaccine, older adults, granzyme B, interferon-gamma, interleukin-10, cytomegalovirus, frailty index

## Abstract

**Background:** Efforts to improve influenza vaccine effectiveness in older adults have resulted in some successes, such as the introduction of high-dose split-virus influenza vaccine (HD-SVV), yet studies of cell-mediated immune responses to these vaccines remain limited. We have shown that granzyme B (GrB) activity in influenza A/H3N2 challenged peripheral blood mononuclear cells (PBMC) correlates with protection against influenza following standard dose vaccination (SD-SVV) in older adults. Further, the interferon-γ (IFNγ) to interleukin-10 (IL-10) ratio can be a correlate of protection.

**Methods:** In a double-blind trial (ClinicalTrials.gov NCT02297542) older adults (≥65 years, *n* = 582) were randomized to receive SD-SVV or HD-SVV (Fluzone®) from 2014/15 to 2017/18. Young adults (20–40 years, *n* = 79) received SD-SVV. At 0, 4, 10, and 20 weeks post-vaccination, serum antibody titers, IFNγ, IL-10, and inducible GrB (iGrB) were measured in *ex vivo* influenza-challenged PBMC. iGrB is defined as the fold change in GrB activity from baseline levels (bGrB) in circulating T cells. Responses of older adults were compared to younger controls, and in older adults, we analyzed effects of age, sex, cytomegalovirus (CMV) serostatus, frailty, and vaccine dose.

**Results:** Prior to vaccination, younger compared to older adults produced significantly higher IFNγ, IL-10, and iGrB levels. Relative to SD-SVV recipients, older HD-SVV recipients exhibited significantly lower IFNγ:IL-10 ratios at 4 weeks post-vaccination. In contrast, IFNγ and iGrB levels were higher in younger SD vs. older SD or HD recipients; only the HD group showed a significant IFNγ response to vaccination compared to the SD groups; all three groups showed a significant iGrB response to vaccination. In a regression analysis, frailty was associated with lower IFNγ levels, whereas female sex and HD-SVV with higher IL-10 levels. Age and SD-SVV were associated with lower iGrB levels. The effect of prior season influenza vaccination was decreased iGrB levels, and increased IFNγ and IL-10 levels, which correlated with influenza A/H3N2 hemagglutination inhibition antibody titers.

**Conclusion:** Overall, HD-SVV amplified the IL-10 response consistent with enhanced antibody responses, with little effect on the iGrB response relative to SD-SVV in either younger or older adults. These results suggest that enhanced protection with HD-SVV is largely antibody-mediated.

**Clinical Trial Registration**: ClinicalTrials.gov (NCT02297542).

## Introduction

Despite widespread vaccination programs, older adults remain susceptible to the serious complications of influenza and experience dramatic increases in hospitalization rates when A/H3N2 is the predominant circulating strain (2017; 2018). Historically, influenza vaccines have been designed to stimulate antibody responses to the critical protective epitopes surrounding the receptor-binding domain of the globular head of hemagglutinin (HA) that permits infection of the host cell. However, although the major protective mechanism of vaccines in young adults is antibody-mediated, older adults also need cytotoxic T lymphocyte (CTL)-mediated clearance of the virus once infection occurs; this provides “clinical protection” against disease in this vulnerable population (Rimmelzwaan and McElhaney, 2008). Nonetheless, when adjusted for the effects of frailty, influenza vaccine effectiveness for the prevention of hospitalization in older adults is similar to that of young adults, suggesting that it is primarily frailty that impacts cellular immunity (Andrew et al., 2017).

Consistent with this notion, prior exposure to influenza through infection or vaccination affects antibody titers and antibody responses to vaccination more so than does aging (Skowronski et al., 2011). In contrast, the decline in cell-mediated immune responses to influenza is related to aging rather than previous exposures to the virus; after age 60, there is a progressive age-related decline in the levels of interferon-γ (IFNγ), IL-10, and the IFNγ:IL-10 ratio secreted by PBMC challenged *ex vivo* with A/H3N2, A/H1N1, or pH1N1 (Skowronski et al., 2011). We have shown that in older adults, vaccination with current inactivated influenza vaccines provides only a weak stimulus to the production of IFNγ by both CD4^+^ and CD8^+^ T cells Kumar et al. (2017) and to the production of the cytolytic mediator, granzyme B (GrB), as well as the generation of cytolytic CD8^+^ T cells in response to *ex vivo* influenza A/H3N2 challenge (McElhaney et al., 2006; McElhaney et al., 2009; Shahid et al., 2010; Zhou and McElhaney, 2011; Haq et al., 2017; Kumar et al., 2017). In contrast, the anti-inflammatory IL-10 response is better preserved and contributes to an overall decline with aging in the IFNγ:IL-10 ratio in response to *ex vivo* influenza challenge (Skowronski et al., 2011). These poor T cell responses have been attributed to the effects of aging [reviewed in (McElhaney et al., 2016; McElhaney et al., 2020a)], persistent cytomegalovirus infection [reviewed in (Merani et al., 2017)], and frailty [reviewed in (McElhaney et al., 2020b)].

Given their role in the cytolysis of infected lung epithelial cells and overall clearance of the virus from the lungs, CD8^+^ CTLs are key to protection against the serious complications of influenza [reviewed in (Altenburg et al., 2015)]. These cells secrete granzyme B (GrB) together with the pore-forming effector, perforin, to enter infected cells and induce apoptosis. Even though only 2–3% of CD8^+^ T cells become positive for both GrB and perforin upon infection, active GrB activity is specifically directed to virus-infected target cells into which it is delivered by perforin; this is a functional readout of physiologically relevant, virus-specific T cell activation (Kula et al., 2019). We have previously shown that GrB activity in *ex vivo* A/H3N2-challenged peripheral blood mononuclear cells (PBMC) correlates with protection against influenza in older adults (McElhaney et al., 2006; McElhaney et al., 2009; Shahid et al., 2010). However, under conditions associated with aging and/or augmented inflammation Hiebert and Granville. (2012), GrB accumulates extracellularly and retains its activity. Thus, in the absence of perforin, GrB is released into the extracellular space and degrades proteins key to maintaining cell-cell junctions Buzza et al.(2005), disrupts epithelial barrier function Matsubara et al. (2020), and increases susceptibility to infection, inflammation, and injury.

We have shown that GrB activity in circulating T cells (bGrB) correlates with the frequency of late-differentiated T cells in older adults and is significantly increased in those seropositive for persistent cytomegalovirus infection relative to those that are seronegative. Upon infection, ∼40% of CD8^+^ T cells become GrB-positive and perforin-negative Kumar et al. (2017) and release GrB (McElhaney et al., 2012). In the extracellular milieu, GrB plays an important role in the loss of tissue function and integrity in the response to an inflammatory stimulus in the lungs (Granville, 2010; Hendel et al., 2010; Kim et al., 2013). This is particularly relevant to acute viral respiratory infection as it has been previously shown that the number of GrB-positive/perforin-negative cells in the lungs and levels of GrB in bronchoalveolar lavage fluid are elevated in patients with acute respiratory distress syndrome Rasmuson et al. (2016) Recently, plasma levels of GrB were identified as one of six key inflammatory analytes in critically ill coronavirus disease 2019 patients (Fraser et al., 2020).

Previous randomized studies comparing HAI antibody and IFNγ-mediated T cell responses to influenza vaccination in older adults have demonstrated the benefit of high-dose (HD) over standard-dose (SD) formulations of seasonal influenza split-virus vaccine (SVV) (Chen et al., 2011; Kim et al., 2016; Cowling et al., 2019). The purpose of our study conducted over four consecutive influenza seasons was to compare antibody, IFNγ, IL-10, and GrB responses to vaccination among older adults randomized to receive the seasonal formulations of HD-SVV vs. SD-SVV, and a young adult control group who received SD-SVV. We have previously reported the antibody responses from this study and showed that frailty was associated with higher titers and increased antibody responses at 4 weeks following influenza vaccination, which was partially dependent on vaccine dosage (Loeb et al., 2020). We now report differences related to age, vaccine dose, CMV serostatus, and frailty in the cell-mediated immune response to influenza vaccination using *ex vivo* PBMC challenged with live influenza A/H3N2.

## Materials and Methods

### Study Design

This study was conducted to compare the cell-mediated immune response to a Fluzone® HD-SVV vs. SD-SVV in community-dwelling older adults against a young adult control group who received Fluzone® SD-SVV [See details at ClinicalTrials.gov: NCT02297542 (Loeb et al., 2020)]. To summarize, a double-blind, re-randomization design, [i.e. participants enrolled in previous years were eligible for enrollment in subsequent years as in the parent trial DiazGranados et al. (2014)] was used to measure pre-vaccination and 4-, 10-, and 20 weeks post-vaccination cell-mediated immune parameters over four influenza seasons (October 2014–April 2015, October 2015–April 2016, October 2016–April 2017, and October 2017–April 2018) as previously described (Loeb et al., 2020). To this end, a total of 582 older adults was enrolled over the four seasons (for years 1–4: 99, 169, 173, and 141, respectively) as previously reported [see Figure 1 in Loeb et al.(2020) and a total of 79 young adults (for years 1–4: 19, 20, 20 and 20, respectively). Those who developed LCII were not excluded from the trial in subsequent years. The study protocol was approved by the Institutional Review Board of the University of Connecticut Health Center and the Health Sciences North Research Ethics Board (Sudbury, ON, Canada) and all study participants provided written informed consent prior to participation in the study.

**FIGURE 1:**
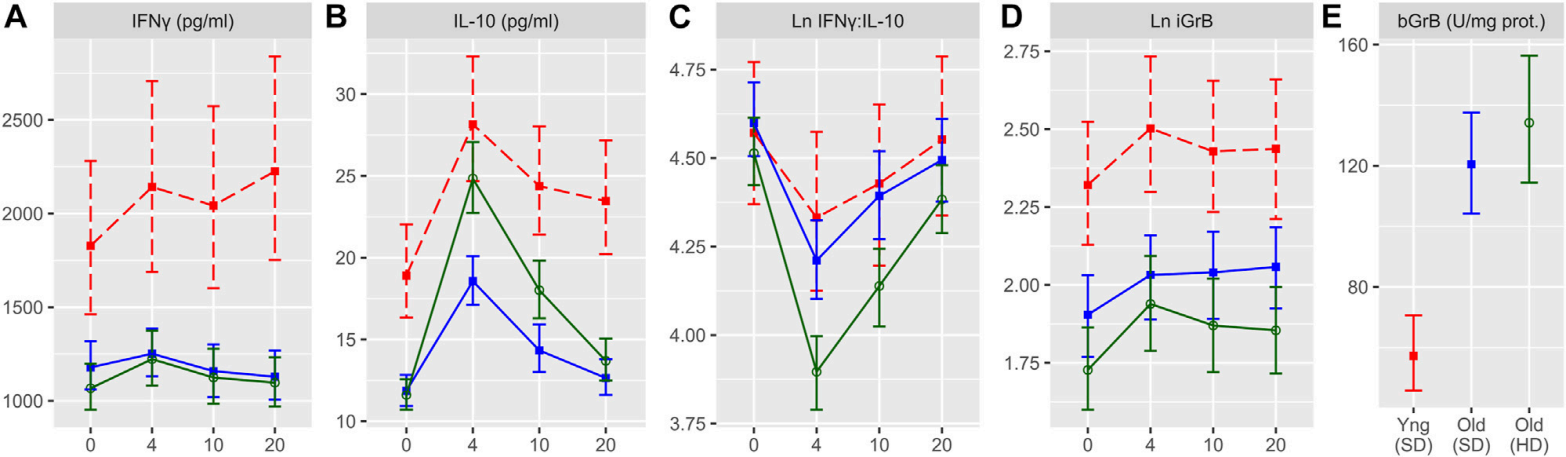
Cell-mediated immune (CMI) responses to live A/H3N2 virus in *ex vivo* stimulated PBMCs for the four study time points (in weeks). IFNγ **(A)**, IL-10 **(B)**, IFNγ:IL-10 ratio **(C)**, and inducible GrB (iGrB levels) **(D)**, and basal GrB (bGrB) **(E)** are presented for young (standard dose; red squares, dashed line) and older (standard dose = blue squares, solid line; high dose = green circles, solid line) adults. The concentration of IFNγ, IL-10 and bGrB are presented as the geometric mean and 95% confidence interval, while the natural log(Ln) IFNγ:IL-10 ratio, and iGrB are presented as the mean and 95% confidence interval.

### Population

Older adults (age 65 years and older) and younger adults (age 20–40 years) were recruited through the University of Connecticut Center on Aging Recruitment Core (UCARC) from communities belonging to and surrounding Hartford, CT, United States, and through the Health Sciences North Research Institute (HSNRI) from the community of Greater Sudbury, ON, Canada. Inclusion criteria included: older adults were at least 65 years old, young adults were 20–40 years old, and all were vaccinated in the previous influenza season. Exclusion criteria were: 1) known immunosuppressive disorders or medications including prednisone in doses >10 mg/day; 2) a previous severe reaction to the vaccine due to egg, latex, or thimerosol allergies, or refusal of vaccination; 3) recipients of influenza vaccination from a community-based program for the approaching influenza season; and 4) pregnancy at week 0/pre-vaccination. Research coordinators ensured that vaccinations were scheduled at least 2 weeks after any acute respiratory illness.

Following informed consent, study participants were characterized according to demographic data [age, sex, ethnicity, and body-mass index (BMI)], chronic medical conditions, including known risk factors for influenza illness (pulmonary, cardiac, metabolic, renal, or neoplastic disorders), health attitudes, symptoms, and functional impairments. A Frailty Index (FI) was calculated based on 40 items validated in terms of outcomes of influenza Theou et al. (2020), which has been previously employed in this trial (Loeb et al., 2020). According to validated cutoffs, people with low FI (<0.10) are robust, those with FI between 0.10 and 0.21 are pre-frail and those with FI > 0.21 are frail. The FI has a well-conserved maximum value of 0.7, (i.e., system failure and death occurs with the accumulation of 2/3 of the total number of deficits) (Hoover et al., 2013).

### Vaccination

Older adult participants were randomized 1:1 to receive either the trivalent, split-virus Fluzone SD vaccine (15 μg of HA per strain) (*n* = 296) or Fluzone HD vaccine (60 μg of HA per strain) (*n* = 286). All young adults received the Fluzone SD vaccine (*n* = 79). This study was conducted over four influenza seasons with the following strains contained in the vaccine each year: 2014/2015: A/California/7/2009 (H1N1)-like virus, A/Texas/50/2012 (H3N2)-like virus, and B/Massachusetts/2/2012-like virus; 2015/2016: A/California/7/2009 (H1N1) pdm09-like virus, A/Switzerland/9715293/2013 (H3N2)-like virus, and B/Phuket/3073/2013-like virus; 2016–2017: A/California/7/2009 (H1N1) pdm09-like virus, A/Hong Kong/4801/2014 (H3N2)-like virus, B/Brisbane/60/2008-like virus; 2017–2018: A/Michigan/45/2015 (H1N1) pdm09-like virus; A/Hong Kong/4801/2014 (H3N2)-like virus; and B/Brisbane/60/2008-like virus. A research nurse not involved in the study administered the vaccine. Clinical and laboratory research staff remained blinded to the vaccine group until all laboratory assays were completed.

### Sample Collection

Blood samples (35 cc heparinized whole blood and 5 cc of serum) were collected at the pre-vaccination and 4-, 10-, and 20 weeks post-vaccination visits. PBMCs were isolated from heparinized blood samples on the day of collection using Ficoll-Plaque Plus (GE Healthcare) gradient purification, frozen in 90% human AB sera and 10% DMSO, and transferred to liquid nitrogen for storage. Plasma and serum samples were collected and stored at −80C.

### Hemagglutination Inhibition Assays

Hemagglutinin-inhibition (HAI) antibody titers were determined at all study time points for each of the three vaccines strains in each year of the study according to standard methodology as previously described (Loeb et al., 2020).

### Cytomegalovirus Serology

CMV serostatus was established using a CMV IgG ELISA kit (Genesis Diagnostics Inc., Cambridgeshire, United Kingdom).

### Cell-Mediated Immune Response Measures

Cell-mediated immune (CMI) measures were performed using our previously described standard operating procedures (SOP) McElhaney and Gentleman. (2015) and validated according to the International Council for Harmonization of Technical Requirements for Pharmaceuticals for Human Use (ICH) (Gijzen et al., 2010). Briefly, thawed PBMCs were stimulated with sucrose-gradient purified live influenza virus (A/Victoria/3/75; Charles River, MA, United States) at a multiplicity of infection of two in AIM V media (Life Technologies, Burlington, ON, Canada) and incubated at 37°C/5%CO_2_ for 20 h. Supernatants and lysates were collected and stored at −80°C until assay measurement. Concentrations of IFNγ and IL-10 were measured in supernatants from A/Victoria/3/75-challenged PBMC by multiplexed bead ELISA (Millipore, Toronto, ON, Canada) and reported as pg/ml. Laboratory testing for cytokine concentration was performed after each study year.

### Granzyme B Assay

The assay of GrB activity has also been validated according to according to the ICH Gijzen et al. (2010) and was performed according to our SOP McElhaney and Gentleman. (2015), which measures enzyme units (U) in a colorimetric assay of the release of paranitroanilade (pna), upon cleavage of the peptide substrate, IEPDpNA (EMD Millipore). GrB activity was adjusted by the total protein content in the cell lysate using the bicinchoninic acid (BCA) assay kit (Pierce, ThermoFisher Scientific), and reported as U/mg of protein (McElhaney and Gentleman, 2015). *Ex vivo* GrB (exGrB) activity was measured in lysates of PBMC that had been challenged for 20 h with A/Victoria/3/75 (H3N2). Basal GrB (bGrB) was determined by measuring GrB activity in lysates of unstimulated CD3^+^ T-cells isolated from thawed PBMC using EasySep T-cell negative selection kits (Stem Cell Technologies). Inducible GrB (iGrB) levels were calculated as iGrB = (exGrB/bGrB) for A/Victoria/3/75 (H3N2).

### Statistical Analysis

Participant demographics were summarized as the mean and standard deviation or count and frequency and HAI and CMI as geometric mean and 95% confidence interval (CI). To estimate the relationship of participant demographics and CMI measures post-vaccination, we employed mixed model linear regression, fitting random intercepts for participant, visit, year, and site. Natural log-transformed CMI measures were scaled to have a mean of zero and standard deviation of one in order to facilitate comparability of regression coefficients across measures, and models were either unadjusted, (i.e. univariate) or adjusted for baseline CMI (matching the dependant CMI measure for a given model), age, sex, vaccine dose, CMV serostatus, and frailty. Separate models were performed for frailty as a categorical and scaled continuous variable, and estimates for baseline CMI are not shown. Results are presented as the coefficient and 95% confidence interval. To estimate the effect of repeat vaccination on scaled CMI or HAI measures post-vaccination, a similar linear mixed model was employed, only year as a continuous variable was included as a fixed effect, in addition to the aforementioned covariates and the previous year’s vaccine dose (participants not enrolled in the previous year were assigned to SD, as per our inclusion criteria). Only vaccination in the immediate previous year was included in the analysis. For models in which the effect of repeat vaccination on the fold-change CMI response, (i.e. week four value vs. baseline) was estimated, the same model was employed, only a random intercept for visit was omitted. Results are presented as the estimated marginal means and 95% confidence interval for each measure considered in each year of study as well as the regression coefficient and *p*-value for the fixed effect of year as a continuous variable. All analyses were conducted using R v3.6.

## Results

### Participants

As previously reported, older adult study participants (*n* = 582) were between 65 and 96 years of age (median, 74), 68% were female, and 49.7% received the HD-SVV. The median frailty index was 0.09 (IQR, 0.051–0.154), with 52 (9%) participants categorized as frail, and 311 (53.4%) participants as CMV-seropositive (Loeb et al., 2020). Characteristics of the three study groups are shown in Table 1. Enrollment and re-enrollment in each study year is summarized in Supplementry Table S1.

**TABLE 1:** Study participant characteristics.

	Older adults (SD) (*N* = 296)	Older adults (HD) (*N* = 286)	Younger adults (SD) (*N* = 79)
Age	74 (69−82)	74 (70−83)	30 (26−33)
Missing	0 (0%)	1 (0.3%)	0 (0%)
Sex			
Female	195 (65.9%)	200 (69.9%)	58 (73.4%)
Male	101 (34.1%)	86 (30.1%)	21 (26.6%)
BMI	28 (24.6−30.5)	27 (24.2−30.4)	25 (23.5−30.7)
Missing	2 (0.7%)	1 (0.3%)	10 (12.7%)
Site			
HSNRI	173 (58.4%)	162 (56.6%)	40 (50.6%)
UCHC	123 (41.6%)	124 (43.4%)	39 (49.4%)
CMV serostatus			
Negative	156 (52.7%)	115 (40.2%)	62 (78.5%)
Positive	140 (47.3%)	171 (59.8%)	17 (21.5%)
Year			
2014/15	49 (16.6%)	50 (17.5%)	19 (24.1%)
2015/16	85 (28.7%)	84 (29.4%)	20 (25.3%)
2016/17	88 (29.7%)	85 (29.7%)	20 (25.3%)
2017/18	74 (25.0%)	67 (23.4%)	20 (25.3%)
Frailty index (continuous)	0.09 (0.0513−0.141)	0.1 (0.0513−0.167)	–
Missing	1 (0.3%)	1 (0.3%)	–
Frailty index (categorical)			
Robust	158 (53.4%)	135 (47.2%)	–
Pre-frail	110 (37.2%)	125 (43.7%)	–
Frail	27 (9.1%)	25 (8.7%)	–
Missing	1 (0.3%)	1 (0.3%)	–

Note: the frailty index was not measured in young adults.

### The Impact of Vaccine Dose on the Response to Influenza Vaccination

In order to examine the impact of vaccine dose and age on levels of cell-mediated immune measures at baseline and post-vaccination, we compared the levels of IFNγ, IL-10, and iGrB in PBMCs challenged *ex vivo* with live H3N2 influenza virus and bGrB levels in resting T cells. Figure 1 shows the change in IFNγ, IL-10, IFNγ:IL-10 ratio, and iGrB levels from pre-to 4 weeks, 10 weeks, and 20 weeks post-vaccination comparing *ex vivo* A/H3N2-challenged PBMCs from younger adults vaccinated with SD-SVV, and older adults vaccinated with HD-SVV or SD-SVV, and adjusted for the effect of prior year vaccination. Overall, IFNγ levels were significantly lower in A/H3N2-challenged PBMCs from older adults, whether they received the SD or HD vaccine, relative to younger adults at all time points (Figure 1A). In contrast, vaccine dose did have a significant impact on IL-10 levels (Figure 1B); prior to vaccination, IL-10 levels were significantly higher in younger than in older adults and peaked at 4 weeks post-vaccination at which point IL-10 levels in older adults vaccinated with HD-SVV were equivalent to those of the younger participants. 4 weeks post-vaccination, IL-10 levels in the older adult group vaccinated with HD-SVV declined to pre-vaccination levels by 20 weeks post-vaccination. This resulted in a significant decrease in the IFNγ:IL-10 ratio at 4 weeks post-vaccination with HD recipients showing the greatest decline compared to younger and older SD recipients (Figure 1C). In all groups, the IFNγ:IL-10 ratio progressively increased 10 weeks and 20 weeks post-vaccination with full or near-full recovery to their pre-vaccination baseline by 20 weeks (Figure 1C). Similar to the IFNγ results, iGrB levels were significantly higher in younger vs. older adults at all time points (Figure 1D). This relates to significantly lower levels of bGrB activity in resting T cells from younger compared to older SD or HD recipients (Figure 1E). In addition, bGrB activity was significantly higher in frail compared to robust older adults (Supplementry Figure S1).

**FIGURE 2:**
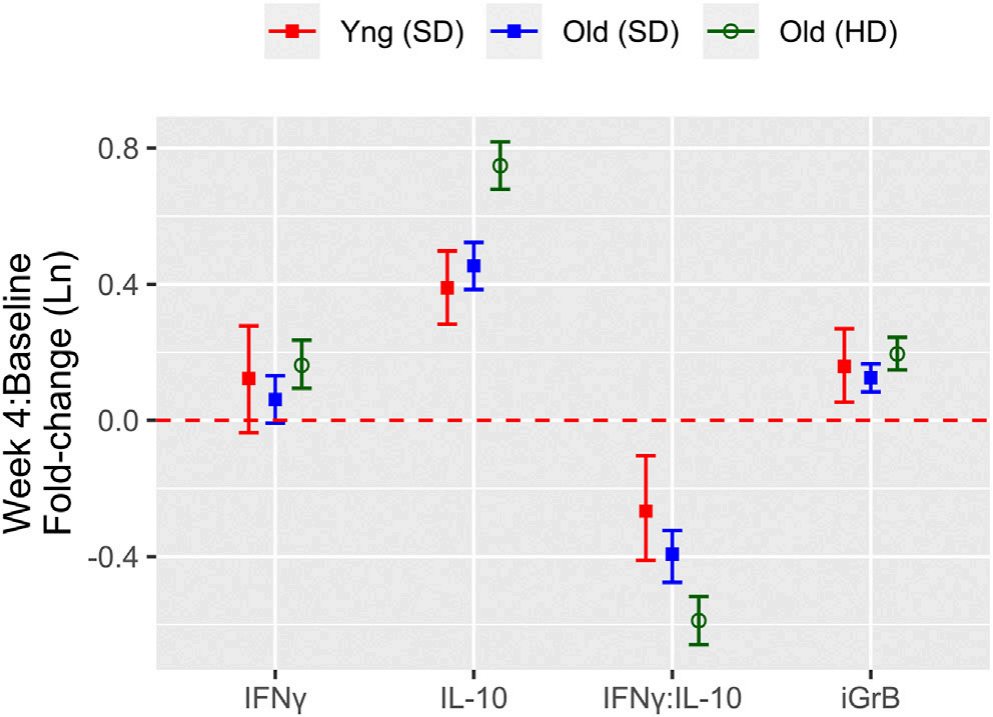
The fold change from pre-to 4 weeks post-vaccination in measures of the cell-mediated immune response to live A/H3N2 virus in *ex vivo* stimulated PBMCs for IFNγ, IL-10, IFNγ:IL-10, and iGrB. The mean and 95% confidence interval of the natural log(Ln) fold-change is presented for young (standard dose; red squares) and older (standard dose = blue squares; high dose = green circles) adults; the red dashed line (fold-change = 0) indicates no response to the vaccine.

The HD-SVV group exhibited a significant increase in IFNγ levels at 4-weeks post-vaccination relative to the pre-vaccination baseline while all groups showed a significant increase of IL-10 and iGrB levels 4 weeks post-vaccination (Figure 2). However, there were significantly lower IFNγ levels at baseline (pre-vaccination) in the HD-SVV vs. SD-SVV groups which may explain the difference in the response to vaccination between the two groups. Along with the increase in IL-10 levels, this difference in baseline IFNγ levels may have contributed to the reciprocal decrease in the IFNγ:IL-10 ratio, which was significantly lower in the older HD recipients than in either the younger or older SD recipients, consistent with a heightened anti-inflammatory response to vaccination (Figure 2). Regulatory T cells (mainly Tr1) as well as B1 cells contained in PBMC cultures secrete IL-10 in large quantities. There was no correlation between the IFNγ:IL-10 ratio and iGrB levels. In contrast, A/H3N2 HAI titers were weakly correlated with IL-10 levels (*r* = 0.185, *p* < 0.001) and this may be related to B1 cells as one of the sources of IL-10 production.

### Effects of Age, Sex, Cytomegalovirus Status, Frailty and Vaccine Dose in Older Adults

**FIGURE 3:**
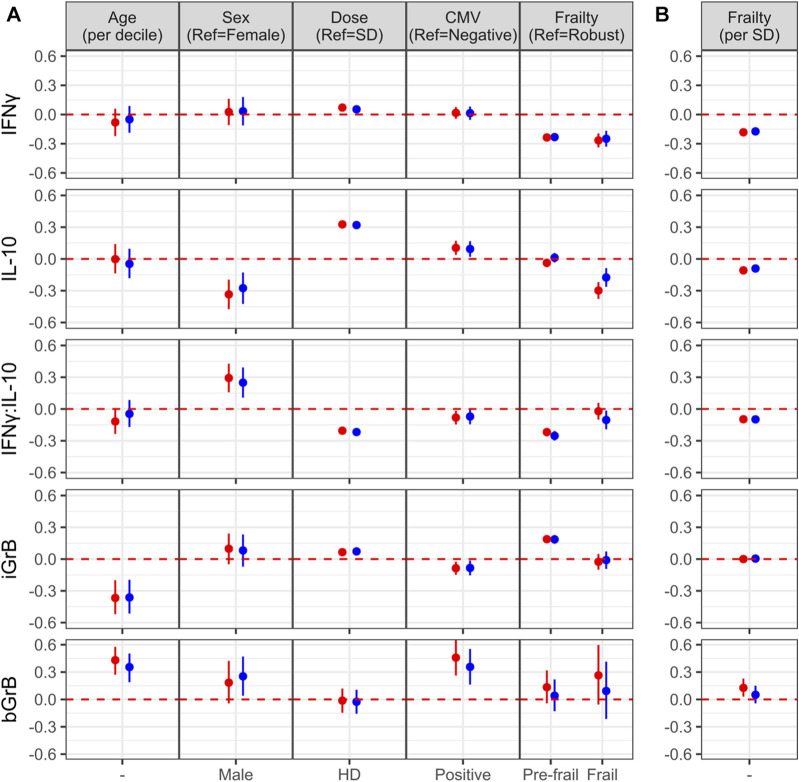
Factors associated with the of cell-mediated immune (CMI) response to vaccination **(A)** The effect of different characteristics of older adult vaccinees are shown for each of the CMI measures in an unadjusted (red dots) and adjusted (blue dots) linear regression analysis, and includes frailty as a categorical variable. For **(B)**, the association between each CMI measure and frailty as standardized continuous variable in both unadjusted and adjusted analyses is presented. The regression coefficient and 95% confidence interval is presented, which represents a 1-standard deviation (SD) change in each measure for a given change in a participant factor, [ie. relative to the reference (ref), or per SD change]. Points and error bars above the red, dashed line indicate a significant positive correlation with a CMI measure, whereas those below the red, dashed line indicate a significant inverse correlation. Note that due to the scale of the *y*-axis, certain confidence intervals are not visible beyond the point estimate.

To determine the effect of age, sex, vaccine dose, CMV serostatus, and frailty on standardized post-vaccination CMI measures (IFNγ, IL-10, iGrB), we performed linear regression on univariate and fully adjusted multivariable models, accounting for the random effect of visit, site, year, and participant; the latter was adjusted for pre-vaccination CMI and all aforementioned factors. In fully adjusted models, IFNγ levels were not significantly affected by age, sex, or CMV, but were significantly higher with HD vaccine [std. *β* (95% CI) = 0.054 (0.029, 0.078)1] and significantly lower in pre-frail [−0.23 (−0.27, −0.19)] and frail [−0.25 (−0.33, −0.17)] vs. robust (non-frail) older adults (Figure 3A). When the level of frailty was considered as a continuous variable using the Frailty Index, increasing levels of frailty correlated with lower levels of IFNγ, where for every 1-standard deviation change in frailty, IFNγ changed by −0.17 standard deviations (95% CI = −0.20, −0.14) (Figure 3B). In contrast, IL-10 levels were significantly lower in males vs. females [std. β (95% CI) = −0.28 (−0.42, −0.13)] and frail vs. robust participants [−0.17 (−0.26, −0.09)] and significantly higher in HD vs. SD vaccine recipients [0.32 (0.29, 0.35)] and CMV-positives [0.094 (0.020, 0.167)] (Figure 3A); IL-10 levels were also significantly, inversely correlated with frailty as a continuous measure [−0.09 (−0.12, −0.056)] (Figure 3B). The IFNγ:IL-10 ratio was significantly increased in males vs. females [std. β (95% CI) = 0.25 (0.11, 0.39)] and significantly lower in HD vs. SD recipients [−0.22 (−0.24, −0.19)] and pre-frail [−0.25 (−0.30, −0.21)] and frail [−0.10 (−0.19, −0.02)] vs. robust older adults (Figure 3A). Frailty as a continuous variable was associated with a lower IFNγ:IL10 ratio, where a 1-SD change in frailty resulted in a 0.10-SD reduction (95% CI = −0.13, −0.064) (Figure 3B).

In contrast to measures of the cytokine response to influenza challenge, only increasing age [std. *β* (95% CI) = −0.36 (−0.51, −0.20)] and CMV-seropositivity [−0.084 (−0.154, −0.015)] was associated with significantly lower iGrB levels, HD vaccine was associated with significantly higher levels [0.072 (0.047, 0.097)] (Figure 3A), while the level of frailty as a continuous variable did not have a significant effect (Figure 3B). Pre-frail vs. robust participants had higher iGrB levels [0.19 (0.14, 0.23)], but not frail vs. robust (Figure 3A). Interestingly, bGrB activity in resting T cells significantly increased with age [std. β (95% CI) = 0.35 (0.19, 0.50)], male sex [0.25 (0.04, 0.47)], and CMV seropositivity [0.36 (0.16, 0.55)] (Figure 3A), while frailty as a continuous variable was not significantly associated (Figure 3B).

**FIGURE 4:**
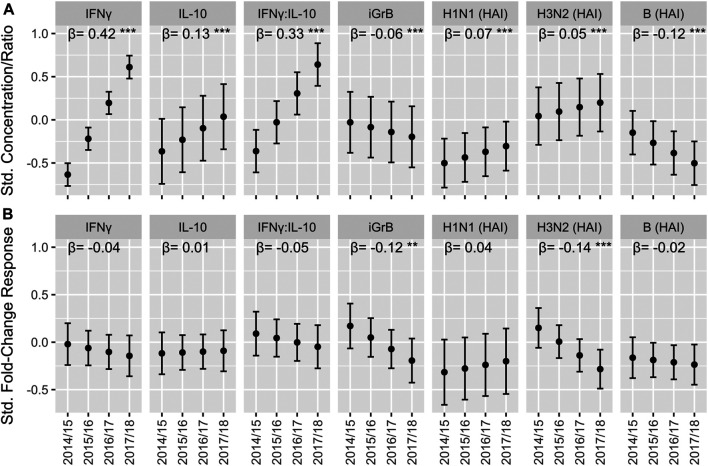
The trend in cell-mediated immune (CMI) responses to live A/H3N2 virus and hemagglutination inhibition (HAI) antibody titers over the course of the study in older adults. For the absolute CMI/HAI measures **(A)** and fold-change in HAI/CMI measures from pre-to 4 weeks post-vaccination **(B)**, the estimated marginal mean and 95% confidence interval of the standardized value (mean = 0, standard deviation = 1) in each year, which was derived from a linear mixed model accounting for repeated measures within participant, is presented on the *y*-axis. The slope (β) and significance (asterisks) of the regression coefficient for year in these models is presented along the top of each plot. ***p < 0.001; **p < 0.01; *p < 0.05.

The effect of prior year vaccination on post-vaccination antibody titers and *ex vivo* CMI responses. There is a body of work suggesting that repeat vaccination may influence antibody responses and thus, vaccine effectiveness (Mosterin Hopping et al., 2016; Belongia et al., 2017). However, this does not take into account the absolute level of immunity achieved following vaccination, (i.e. level of seroprotection or CMI). Thus, in a post-hoc analysis, we analyzed the overall effect of vaccination on both post-vaccination antibody titers and *ex vivo* CMI responses in older adults over the course of our trial without distinguishing between prior year and current year vaccination subsets, (i.e. HD-HD, HD-SD, SD-HD, and SDSD) in this analysis. We found that the absolute levels of IFNγ [Std. β (95% CI) = 0.42 (0.40, 0.43)], IL-10 [0.13 (0.11, 0.15)] and the IFNγ:IL-10 ratio [0.33 (0.32, 0.35)] increased significantly with each study year, while iGrB levels exhibited a smaller but statistically significant decline [−0.056 (−0.077, −0.037)] (Figure 4A). In contrast, absolute hemagglutination inhibition (HAI) antibody titers to A/H1N1 [Std. *β* (95% CI) = 0.065 (0.048, 0.085)] and A/H3N2 [0.052 (0.029, 0.072)] strains increased significantly while HAI titers to influenza B declined significantly over the four years [−0.12 (−0.14, −0.10)] (Figure 4A). The fold-change in H3N2 iGrB levels [Std. β (95% CI) = −0.12 (−0.20, −0.04)] and H3N2 HAI titers [−0.14 (−0.23, −0.06)] from pre-to 4-weeks post-vaccination significantly declined over the four years, while there were no significant changes in the other response measures (Figure 4B). As expected, bGrB levels did not change significantly from year to year (data not shown).

## Discussion

Based on the humoral response, several influenza vaccine formulations have demonstrated enhanced immunogenicity Nicolay et al.(2019) and superior efficacy over standard dose comparator vaccines in randomized trials in older adults (DiazGranados et al., 2014; Dunkle et al., 2017). However, there have been few randomized trials that have compared cytokine-mediated immune responses to these approved enhanced influenza vaccines against a standard dose comparator vaccine in older adults Kumar et al.(2017); Cowling et al.(2019), and none have studied the cytolytic T cell response. To this end, we conducted a randomized trial of HD-SVV vs. SD-SVV over 4 years. The primary comparison planned was between those older adults randomized to the HD vs. SD formulation of seasonal influenza vaccine using our established methods for measuring cytokine (IFNγ, IL-10) and cytolytic T cell (iGrB) responses to vaccination. We found that HD-SVV significantly increased the IFNγ response (produced by both CD4^+^ and CD8^+^ T cells) to vaccination but this result may have been affected by differences in the baseline levels of IFNγ (HD-SVV < SD-SVV) and may explain why there was no difference in the iGrB response to vaccination in HD-SVV recipients when compared to younger and older SD-SVV recipients. Thus, HD-SVV would not be expected to provide additional benefit over SD-SVV in the stimulation of cytolytic CD8 T cells needed to clear the virus from the lungs. In contrast to the GrB activity detected in unstimulated T cells (bGrB), our unpublished data has shown that there are no detectable levels of IFNγ or IL-10 in the supernatants of unstimulated T cells. Further, GrB activity in unstimulated PBMC is largely derived from T cells and thus we have used bGrB activity (in isolated unstimulated T cells) to adjust for this effect in *ex vivo* challenged PBMC.

Previous randomized trials have measured the CMI response to influenza vaccination in older adults according to changes in IFNγ^+^, CD4^+^, or CD8^+^ T cell frequencies (Cowling et al., 2019; Pillet et al., 2019). Although these IFNγ^+^ T cell frequencies have been shown to be a correlate of protection against influenza A in young adults with low antibody titers, the *ex vivo* response is specific for peptides derived from the internal proteins, matrix (M1), and nucleoprotein (NP), which are shared between influenza A/H1N1 and A/H3N2 strains but distinct from those of influenza B (Wilkinson et al., 2012; Sridhar et al., 2013). This becomes important when considering the content of the vaccine: SVV formulations studied in this trial contain significant quantities of M1 and NP Co et al.(2009) while subunit Kumar et al.(2017) and recombinant HA formulations contain no internal proteins. In addition, we have shown that IL-10 is an important mediator of the response to vaccination; a low IFNγ:IL-10 ratio in A/H3N2-challenged PBMC following vaccination with SD-SVV is an important predictor of risk for febrile LCII in older adults (Shahid et al., 2010). The production of IL-10 may curtail undesirable inflammatory responses to A/H3N2 infection simulated in A/H3N2-challenged PBMC. In a randomized, double-blind, placebo-controlled trial, we showed that influenza vaccination attenuated the inflammatory response in patients undergoing cardiopulmonary bypass surgery; there was a reduction in serum levels of troponin (as a measure of myocardial injury) and pro-inflammatory mediators [IL-6, IL-8, tumor necrosis factor (TNF), C-reactive protein], and an increase in IL-10 (Atoui et al., 2020). However, given the kinetics of the IL-10 response, which peaks at 4-weeks vaccination, this response as a correlate of protection appears to vary depending on timing of vaccination relative to the onset of the influenza season (McElhaney et al., 2006; Shahid et al., 2010).

The level of GrB activity measured in *ex vivo* A/H3N2 PBMC (exGrB) reflects the activation of the 2–3% of CD8^+^ T cells that are GrB^+^Perforin^+^ and the 40% of CD8^+^ T cells that are GrB^+^Perforin− (Kumar et al., 2017). We have previously shown that the *ex vivo* activation of influenza-specific memory T cells corresponds to the cytolytic activity of GrB^+^Perforin^+^ CD8^+^ T cells, which is reduced in both magnitude and duration of the response to SD-SVV in older adults relative to the young (Zhou and McElhaney, 2011). In contrast, GrB^+^Perforin− CD8^+^ T cells degranulate in response to *ex vivo* influenza challenge McElhaney et al.(2012) and thus have no influenza-specific cytolytic activity in the absence of perforin, while also contributing to local tissue damage. We have therefore developed an assay of bGrB activity in circulating T cells that correlates with the proportion of late/terminally differentiated CD8^+^ T cells (Haq et al., 2017). These GrB^+^Perforin^**-**^ CD8^+^ T cells are present in the circulating T cell pool McElhaney et al.(2012) and contribute to basal levels of GrB (bGrB) activity which are: 1) significantly higher in CMV-seropositive vs. CMV-seronegative older adults; 2) positively correlated with the frequency of late/terminally differentiated CD8^+^ T cells; and 3) negatively correlated with the frequency of memory CD8^+^ T cells (Haq et al., 2017). The potential limitations of this analysis of the cell-mediated immune response to influenza vaccination include the absence of measures of different T cell subset frequencies that we have correlated with the cytolytic activity of CD8^+^ T cells Zhou and McElhaney,(2011) and other studies demonstrating that IFNγ^+^, CD4^+^, and CD8^+^ T cells responding to M1 and NP peptides are correlates of protection against influenza (Wilkinson et al., 2012; Sridhar et al., 2013).

To adjust for the contribution of GrB activity presumably derived from the non-specific inflammatory response to *ex vivo* A/H3N2 challenge in late/terminally differentiated CD8^+^ T cells, we calculated iGrB levels as exGrB activity divided by bGrB activity. We observed the expected age-related decline in the iGrB response to *ex vivo* challenge both pre- and post-vaccination. In the absence of this adjustment, exGrB activity was highest in frail older adults and lowest in younger adults. Taken together, these results suggest that bGrB activity accounts for the effect of age and increasing frailty in the iGrB response to *ex vivo* influenza challenge. Consistent with this finding, all three vaccination groups showed a significant increase in iGrB levels 4 weeks post-vaccination. There was a trend toward a higher fold-increase in iGrB levels 4-weeks post-vaccination in HD vs. SD recipients; the overall weak stimulus to memory CD8 T cells provided by inactivated influenza vaccines may explain why this did not reach statistical significance.

In the regression analysis, we found that increasing age and CMV seropositivity are significant correlates of the decreased iGrB response to *ex vivo* influenza challenge. CMV has been shown to be the main driver of oligoclonal expansions of CD8^+^ T cells with altered phenotypes and functions (Pawelec et al., 2005). As a component of the Immune Risk Phenotype, CMV seropositivity is an important predictor of mortality (Pawelec et al., 2005). We have since shown that bGrB activity is increased in CMV seropositive vs. seronegative older adults and correlates with the frequency of late/terminally differentiated CD8^+^ T cells that result from oligoclonal expansion. Using the Frailty Index, which is also a strong predictor of mortality Clegg et al.(2013), we now show that although bGrB activity increases with age and increased levels of frailty, CMV seropositive status is a significant contributor to this relationship. In the extracellular space, GrB contributes to enhanced inflammatory responses Hiebert and Granville,(2012) that may suppress the response influenza vaccination. In addition, increased frailty is associated with a lower IFNγ response to *ex vivo* A/H3N2 challenge. Our previous studies suggest that the addition of a toll-like receptor (TLR4) agonist to SVV can overcome the suppressive effects of IL-10 and improve the IFNγ response and markedly improve the IFNγ:IL-10 ratio and GrB response to influenza challenge (Behzad et al., 2012).

In light of the current controversy over the effects of repeated annual influenza vaccination potentially leading to a loss of vaccine-mediated protection against influenza, we analyzed the effect of vaccination in each study year according to the absolute levels of antibody, cytokines (IFNγ, IL-10, and IFNγ:IL-10) and iGrB across the four study time points and the pre-to 4 weeks post-vaccination response in older adults. We found a significant decline only in the A/H3N2 iGrB and antibody response to influenza vaccination. Our previous study showed that this decline in antibody responses to influenza vaccination in older adults is an effect of repeated vaccination and not age (Mosterin Hopping et al., 2016). In contrast, absolute antibody titers and cytokine levels across the four study time points, increased with each successive year of vaccination in the study. This is important because previous studies have shown that there is no significant impact of prior-season vaccination on vaccine effectiveness for the prevention of influenza hospitalizations in older adults (Cheng et al., 2017; Song et al., 2020). Consistent with our iGrB results showing a decline in the iGrB levels with prior season vaccination, Nichols et al reported that prior season influenza vaccination resulted in a non-significant decline in vaccine effectiveness for the prevention of hospitalization. However, prior season vaccination was still more effective than not receiving the current season’s vaccine (Nichols et al., 2019). These results suggest that the decline in iGrB levels with prior season influenza vaccination correlates with protection against hospitalization but this decline in iGrB levels does not appear to be clinically significant. Further, these findings highlight the importance of re-stimulating the iGrB response with annual influenza vaccination. Relevant to our clinical trial, it has been previously shown in a large clinical trial of HD-SVV vs. SD-SVV in older adults over two influenza seasons where A/H3N2 was the predominant circulating strain, that vaccination with HD-SVV compared to SD-SVV was associated with lower risk of LCII irrespective of prior-season vaccination (DiazGranados et al., 2016). Thus, we are planning to analyze the effect of prior-season vaccination on all of our immunologic measures included in our trial.

The strengths of the study are the kinetic analysis of the response to vaccination and also the study of immune responses in the context of prior season vaccination. However, there are important limitations to the study. The first limitation of this study relates to the recruitment of a relatively younger segment of the over 65 population. Thus, these results may not extend to adults age 85 years and older as is often the case in randomized trials of influenza vaccines. Even so, half of the sample were pre-frail or frail, suggesting that the trial participants were not limited to fit younger seniors. In addition, the trial was not designed to study the effect of repeat vaccination and thus we were limited to studying the effect of prior year vaccination without distinguishing between HD vs. SD vaccines.

The second limitation is that we have not accounted for the contribution of NK cells to the total amount of GrB activity measured in *ex vivo* A/H3N2 challenged PBMC. Our unpublished data has shown that with *ex vivo* A/H3N2 challenge, there is a 40-fold increase of GrB activity in isolated T cells, most of which is derived from CD8^+^ T cells. In contrast, a recent study showed that the NK cell response to influenza infection resulted in a 2-fold increase in the Mean Fluorescence Intensity (MFI) of intracellular GrB (with no change in the MFI for perforin) in NK cells (Scharenberg et al., 2019). Further, NK cells contribute to GrB activity in influenza-stimulated PBMC but the co-expression of Perf (needed for cytolytic activity) in these NK cells has been shown to be T cell dependent (He et al., 2004). We have shown that older compared to young adults have an enhanced GrB^+^ CD8^+^ T cell response to *ex vivo* A/H3N2 challenge when compared to unstimulated PBMC, whereas the GrB^+^ NK cell response is similar in young and older adults (McElhaney et al., 2009). The age-related increase in the GrB^+^ CD8^+^ T cell response appears to be related GrB^+^ CD8^+^ T cells that do not co-express Perf. Taken together, these results suggest that the cytolytic potential based on the amount of GrB produced on a per cell basis in response to *ex vivo* challenge is much greater in CD8^+^ T cells compared to NK cells but further studies are needed to understand how aging may affect the cytolytic potential (GrB^+^Perf^+^) vs. potential damaging effects (GrB^+^Perf^−^) of CD8^+^ T cells and NK cells.

A third limitation inherent to the study design is that the source of the cytokines cannot be identified. B1 cells, regulatory T cells and NK cells which are part of PBMC cultures, may also produce the cytokines measured. A more detailed analysis using intracellular cytokine staining and flow cytometric analysis of *ex vivo* stimulated PBMC is needed to resolve this issue. However, we have already found that IL-10-producing T cells are not detectable using standard methods for intracellular cytokine staining.

In summary, this study highlights the age-related decline in the IFNγ and iGrB response to influenza challenge which corresponds to the increased risk for serious complications of influenza in older adults. These responses are only weakly stimulated by SVV, independent of vaccine dose. In contrast, HD-SVV amplified the IL-10 response, with little effect on the iGrB response relative to SD-SVV in either younger or older adults. Thus, the benefit of HD over SD vaccines in older adults may be largely antibody-mediated but not necessarily limited to the neutralizing effects measured in hemagglutination inhibition assays. In light of these results, our future direction is to characterize the IFNγ and iGrB responses to vaccination according to the frequency of different CD4^+^ and CD8^+^ T cell subsets and understand how inflammatory (IL-6, IL-8, and TNF) and anti-inflammatory cytokines (IL-10) produced in *ex vivo* challenged PBMC, affect the frequency of these T cell subsets. In addition, we want to advance our studies of the effect of prior season vaccination to study the effect of repeated annual influenza vaccination. Ultimately, these biomarkers of protection could be incorporated into early phase clinical trials in the development of more effective influenza vaccines for older adults.

## Data Availability

The raw data supporting the conclusion of this article will be made available by the authors, without undue reservation.
