# Corrigendum: Key Determinants of Cell-Mediated Immune Responses: A Randomized Trial of High Dose Vs. Standard Dose Split-Virus Influenza Vaccine in Older Adults

**DOI:** 10.3389/fragi.2021.718966

**Published:** 2021-06-25

**Authors:** Chris P. Verschoor, Laura Haynes, Graham Pawelec, Mark Loeb, Melissa K. Andrew, George A. Kuchel, Janet E. McElhaney

**Affiliations:** ^1^ Health Sciences North Research Institute, Sudbury, ON, Canada; ^2^ Northern Ontario School of Medicine, Sudbury, ON, Canada; ^3^ UConn Center on Aging, University of Connecticut School of Medicine, Farmington, CT, United States; ^4^ Department of Immunology, University of Tübingen, Tübingen, Germany; ^5^ Department of Pathology and Molecular Medicine, McMaster University, Hamilton, ON, Canada; ^6^ Department of Medicine (Geriatrics), Dalhousie University, Halifax, NS, Canada

**Keywords:** cell-mediated immune responses, high-dose vs. standard-dose split-virus influenza vaccine, older adults, granzyme B, interferon-gamma, interleukin-10, cytomegalovirus, frailty index

In the original article, there was a mistake in Figures 3 and 4 as published. A coding error with the statistical analysis led to erroneous estimates, mainly for associations with the frailty index. Although all estimates were altered slightly, the overall conclusion was not impacted greatly. The corrected Figures 3 and 4 appear below.

**FIGURE 3:**
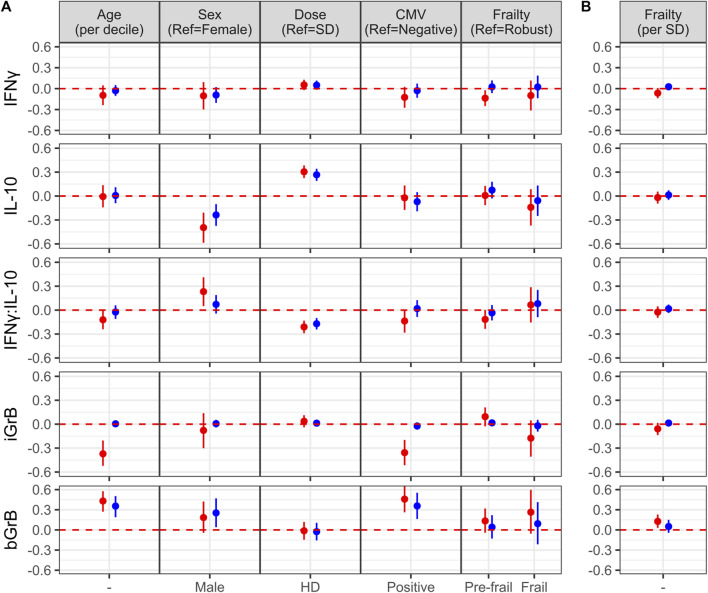
Factors associated with the of cell-mediated immune (CMI) response to vaccination. **(A)** The effect of different characteristics of older adult vaccinees are shown for each of the CMI measures in an unadjusted (red dots) and adjusted (blue dots) linear regression analysis, and includes frailty as a categorical variable. For **(B)**, the association between each CMI measure and frailty as standardized continuous variable in both unadjusted and adjusted analyses is presented. The regression coefficient and 95% confidence interval is presented, which represents a 1-standard deviation (SD) change in each measure for a given change in a participant factor, [ie., relative to the reference (ref), or per SD change]. Points and error bars above the red, dashed line indicate a significant positive correlation with a CMI measure, whereas those below the red, dashed line indicate a significant inverse correlation. Note that due to the scale of the *y*-axis, certain confidence intervals are not visible beyond the point estimate.

**FIGURE 4:**
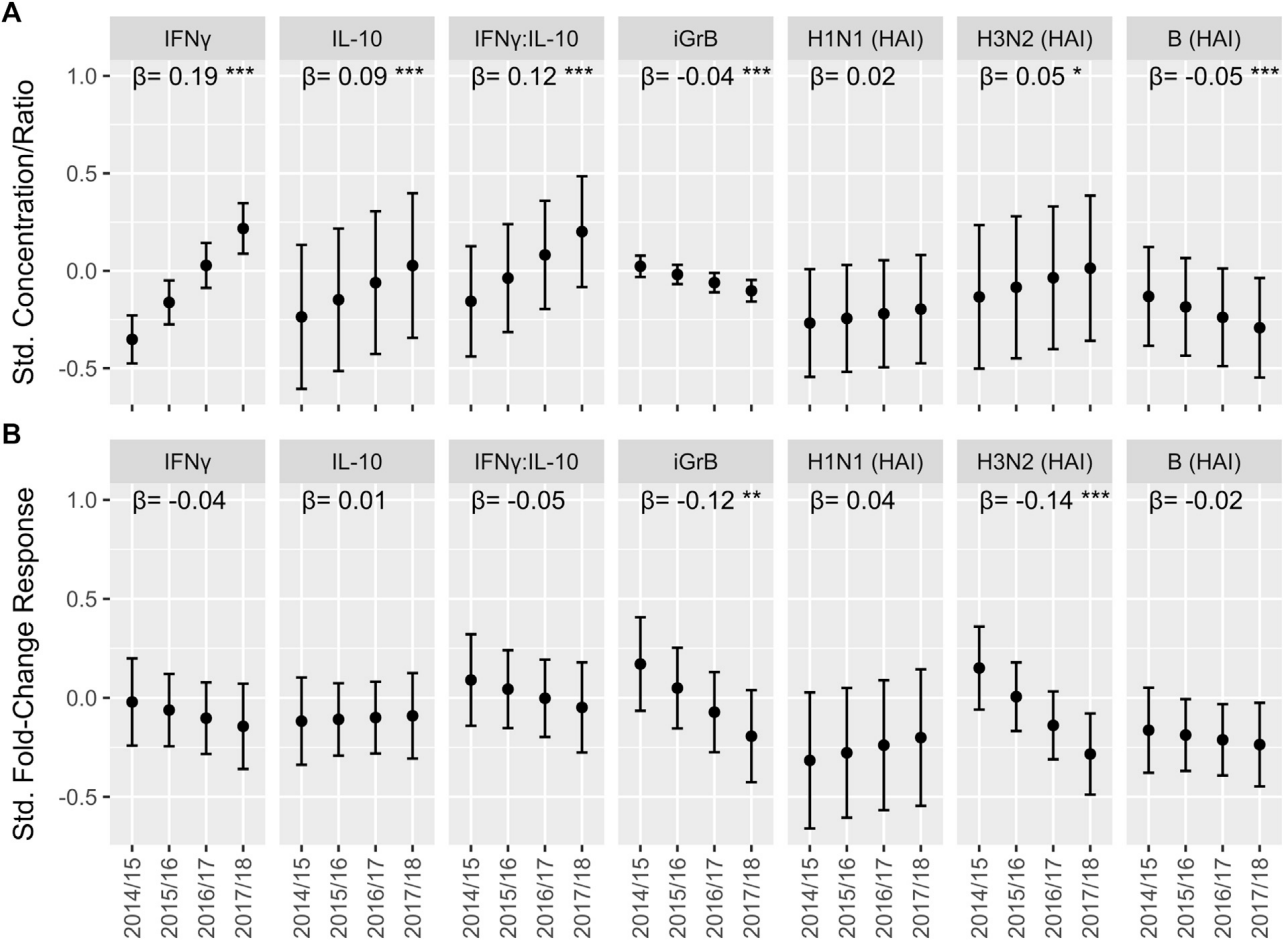
The trend in cell-mediated immune (CMI) responses to live A/H3N2 virus and hemagglutination inhibition (HAI) antibody titers over the course of the study in older adults. For the absolute CMI/HAI measures **(A)** and fold-change in HAI/CMI measures from pre-to 4 weeks post-vaccination **(B)**, the estimated marginal mean and 95% confidence interval of the standardized value (mean 0, standard deviation 1) in each year, which was derived from a linear mixed model accounting for repeated measures within participant, is presented on the *y*-axis. The slope (β) and significance (asterisks) of the regression coefficient for year in these models is presented along the top of each plot. ***p < 0.001; **p < 0.01; *p < 0.05.

Due to the coding error in the statistical analysis, the description of the findings presented in the **Abstract**, **Results**, and **Discussion** could also be misleading.

A change was made in the **Abstract**, sub-section “Results,” Line 6. The corrected line now reads: “In a regression analysis, female sex and HD-SVV were associated with higher IL-10 levels, while SD-SVV was associated with lower iGrB levels.”

A change was also made in the **Results**, sub-section “Effects of Age, Sex, Cytomegalovirus Status, Frailty and Vaccine Dose in Older Adults,” Paragraphs 1–3. The corrected text appears below:

## Results

Effects of Age, Sex, Cytomegalovirus Status, Frailty and Vaccine Dose in Older Adults

To determine the effect of age, sex, vaccine dose, CMV serostatus and frailty on standardized post-vaccination CMI measures (IFNγ, IL-10, iGrB), we performed linear regression on univariate and fully-adjusted multivariable models, accounting for the random effect of visit, site, year and participant; the latter was adjusted for pre-vaccination CMI and all aforementioned factors. In fully-adjusted models, IFNγ levels were not significantly affected by age, sex, CMV, dose or frailty (Figure 3). In contrast, IL-10 levels were significantly lower in males vs. females (std. β [95% CI] = −0.24 [−0.37, −0.10]) and significantly higher in HD vs. SD vaccine recipients (0.27 [0.19, 0.34]) (Figure 3A). The IFNγ:IL-10 ratio was significantly lower in HD vs. SD recipients (std. β [95% CI] = −0.17 [−0.24, −0.10]) (Figure 3A). In contrast to measures of the cytokine response to influenza challenge, none of the factors studied affected iGrB levels post-vaccination (Figure 3). Interestingly, bGrB activity in resting T cells significantly increased with age (std. β [95% CI] = 0.35 [0.19, 0.50]), male sex (0.25 [0.04, 0.47]), and CMV seropositivity (0.36 [0.16, 0.55]) (Figure 3A), while frailty as a continuous variable was not significantly associated (Figure 3B).

We found that the absolute levels of IFNγ (Std. β [95% CI] = 0.19 [0.15, 0.23]), IL-10 (0.09 [0.05, 0.13]) and the IFNγ:IL-10 ratio (0.12 [0.08, 0.16]) increased significantly with each study year, while iGrB levels exhibited a smaller but statistically significant decline (−0.042 [−0.059, −0.025]) (Figure 4A). In contrast, absolute hemagglutination inhibition (HAI) antibody titers to A/H3N2 (0.049 [0.005, 0.091]) strains increased significantly while HAI titers to influenza B declined significantly over the 4 years (−0.054 [−0.084, −0.023]) (Figure 4A).

## Discussion

Finally, a change was made to the **Discussion**, Paragraph 5, Line 1, which now reads: “In univariate regression analysis, we found that increasing age and CMV seropositivity are significant correlates of the decreased iGrB response to *ex vivo* influenza challenge.”

Paragraph 5, Line 19 now reads: “Our previous studies suggest that the addition of a toll-like receptor (TLR4) agonist to SVV can overcome the suppressive effects of IL-10 and improve the GrB response to influenza challenge (Behzad et al., 2012).”

A change was also made in Paragraph 6, Line 1, which now reads: “In light of the current controversy over the effects of repeated annual influenza vaccination potentially leading to a loss of vaccine-mediated protection against influenza, we analyzed the effect of vaccination in each study year according to the absolute levels of antibody, cytokines (IFNγ, IL-10, and IFNγ:IL-10) and iGrB at 4-, 10-, and 20-weeks post-vaccination and the pre- to 4-weeks post-vaccination fold-change response in older adults. We found a significant decline over the 4 study years only in the post-vaccination A/H3N2 iGrB levels and influenza B antibody titers.”

The authors apologize for these errors and state that this does not change the scientific conclusions of the article in any way. The original article has been updated.

