# Supplementary material for: Key Determinants of Cell-Mediated Immune Responses: A Randomized Trial of High Dose Vs. Standard Dose Split-Virus Influenza Vaccine in Older Adults
Source: Front Aging. 2021 May 21;2:649110. doi: 10.3389/fragi.2021.649110 (PMC8813165; doi:10.3389/fragi.2021.649110)
Supplement: Supplementary file 1 [file DataSheet1.docx]

Supplemental Table 1. Older adult participant enrollment in each study year

|  | SD* | HD** | Total | Number in previous year | Number in any other year |
| --- | --- | --- | --- | --- | --- |
| Year 1 | 49 | 50 | 99 | 0 | 81 |
| Year 2 | 85 | 84 | 169 | 77 | 152 |
| Year 3 | 88 | 85 | 173 | 136 | 156 |
| Year 4 | 74 | 67 | 141 | 117 | 122 |

*Standard Dose Vaccine (SD) and **High Dose Vaccine (HD)

**
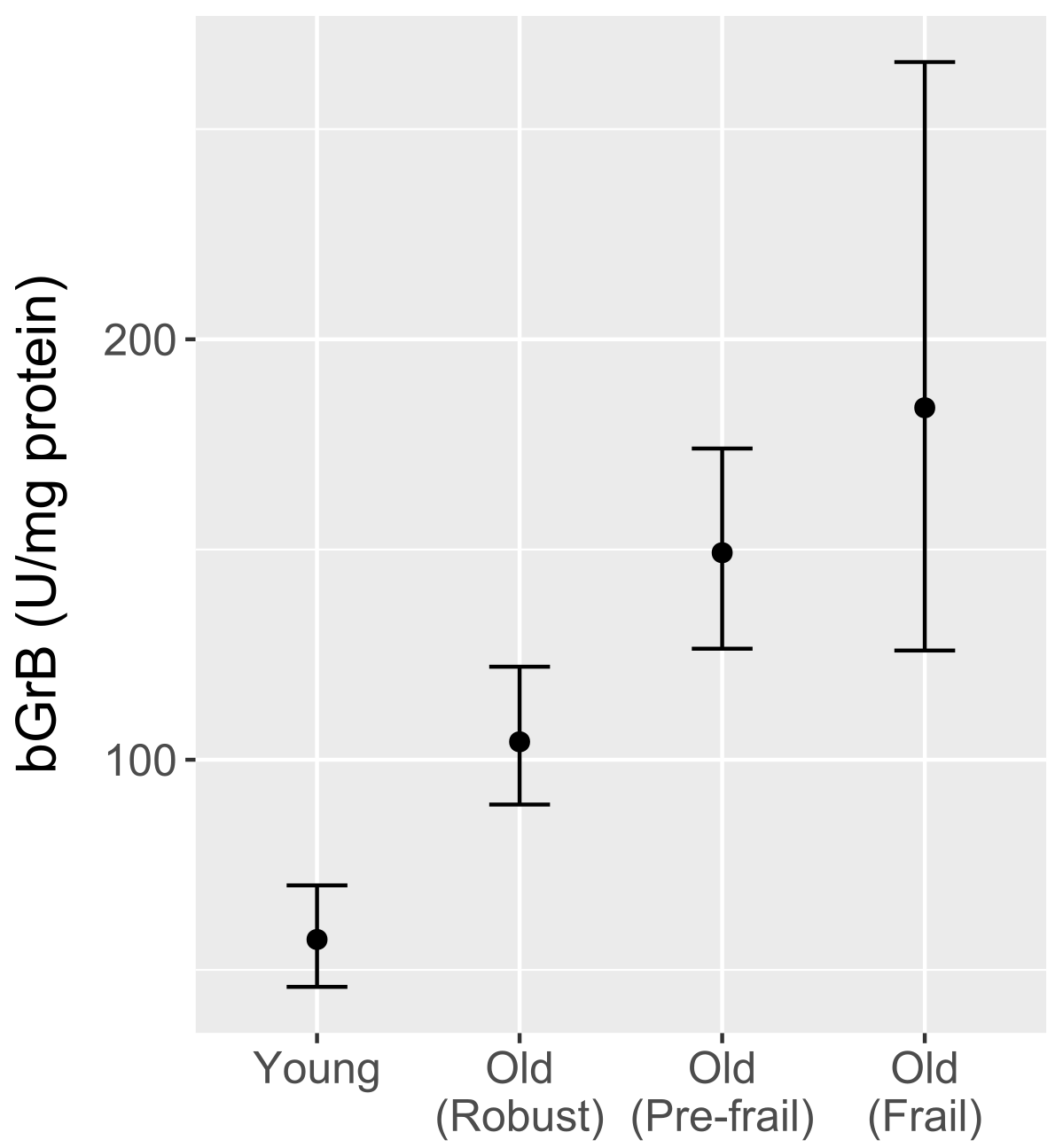
**

**Supplemental Figure 1.** Granzyme B (GrB) activity was measured in T cells isolated from thawed PBMC and represent basal GrB (bGrB) activity in circulating T cells. The geometric mean and 95% CI bGrB activity is shown for each of the young adult and robust, pre-frail and frail subsets of older adults in the study.
